# Supplementary material for: Identification of new drugs to counteract anti-spike IgG-induced hyperinflammation in severe COVID-19
Source: Life Sci Alliance. 2023 Sep 12;6(11):e202302106. doi: 10.26508/lsa.202302106 (PMC10497933; doi:10.26508/lsa.202302106)
Supplement: Supplementary file 1 [file LSA-2023-02106_TableS1.docx]

**Table S1**

| **Name** | **Affiliations** |
| --- | --- |
| M. A. van Agtmael | Department of Infectious Diseases |
| A. G. Algera | Department of Intensive Care |
| B. Appelman | Department of Infectious Diseases |
| F. E. H. P. van Baarle | Department of Intensive Care |
| D. van de Beek | Department of Neurology |
| M. Beudel | Department of Neurology |
| H. J. Bogaard | Department of Pulmonology |
| M. Bomers | Department of Infectious Diseases |
| P.I. Bonta | Department of Pulmonology |
| L. D. J. Bos | Department of Intensive Care |
| M. Botta | Department of Intensive Care |
| J. de Brabander | Department of Infectious Diseases |
| G. J. de Bree | Department of Infectious Diseases |
| M. C. Brouwer | Department of Neurology |
| S. de Bruin | Department of Intensive Care |
| M. Bugiani | Department of Pathology |
| E. B. Bulle | Department of Intensive Care |
| O. Chouchane | Department of Infectious Diseases |
| A. P. M. Cloherty | Experimental Immunology |
| D. Buis | Department of Infectious Diseases |
| M. C. F. J. de Rotte | Department of Clinical Chemistry |
| M. Dijkstra | Department of Clinical Chemistry |
| D. A. Dongelmans | Department of Intensive Care |
| R. W. G. Dujardin | Department of Intensive Care |
| P.E. Elbers | Department of Intensive Care |
| L. M. Fleuren | Department of Intensive Care |
| S. E. Geerlings | Department of Infectious Diseases |
| T. B. H. Geijtenbeek | Department of Experimental Immunology |
| A. R. J. Girbes | Department of intensive care |
| A. Goorhuis | Department of Infectious Diseases |
| M. P. Grobusch | Department of Infectious Diseases |
| L. A. Hagens | Department of Intensive Care |
| J. Hamann | [Amsterdam UMC Biobank Core Facility](mailto:j.hamann@amsterdamumc.nl) |
| V. C. Harris | Department of Infectious Diseases |
| R. Hemke | Department of Radiology |
| S. M. Hermans | Department of Infectious Diseases |
| L. M. A. Heunks | Department of Intensive Care |
| M. W. Hollmann | Department of Anesthesiology |
| J. Horn | Department of Intensive Care |
| J. W. Hovius | Department of Infectious Diseases |
| M. D. de Jong | Department of Medical Microbiology |
| R. Koing | Department of Neurology |
| E. H. T. Lim | Department of Intensive Care |
| N. van Mourik | Department of Intensive Care |
| J. F. Nellen | Department of Infectious Diseases |
| E. J. Nossent | Department of Pulmonology |
| F. Paulus | Department of Intensive Care |
| E. Peters | Department of Infectious Diseases |
| D. Piña-Fuentes | Department of neurology |
| T. van der Poll | Department of Infectious Diseases |
| B. Preckel | Department of Anesthesiology |
| S .J. Raasveld | Department of Intensive Care |
| T. D.Y. Reijnders | Department of Infectious Diseases |
| M. Schinkel | Department of Infectious Diseases |
| F. A.P. Schrauwen | Department of Clinical Chemistry |
| M. J. Schultz | Department of Intensive Care |
| A. R. Schuurman | Department of Internal Medicine |
| J. Schuurmans | Department of Intensive Care |
| K. Sigaloff | Department of Infectious Diseases |
| M. A. Slim | Department of Intensive Care and Infectious Diseases |
| P. Smeele | Department of Pulmonology |
| M. R. Smit | Department of Intensive Care |
| C. Stijnis | Department of Infectious Diseases |
| W. Stilma | Department of Intensive Care |
| C. E. Teunissen | Neurochemical Laboratory |
| P. Thoral | Department of Intensive Care |
| A. M. Tsonas | Department of Intensive Care |
| P. R. Tuinman | Department of Intensive Care |
| M. van der Valk | Department of Infectious Diseases |
| D. P. Veelo | Department of Anesthesiology |
| A. P. J. Vlaar | Deparment of Intensive Care |
| C. Volleman | Department of Intensive Care |
| H. de Vries | Department of Intensive Care |
| L. A. van Vught | Department of Intensive Care and Infectious Diseases |
| M. van Vugt | Department of Infectious Diseases |
| W. J. Wiersinga | Department of Infectious Diseases |
| D. Wouters | Department of Clinical Chemistry |
| A. H. Zwinderman | Department of Clinical Epidemiology, Biostatistics and Bioinformatics |
